# Supplementary material for: Evaluation of dietary selenium methionine levels and their effects on growth performance, antioxidant status, and meat quality of intensively reared juvenile Hypophthalmichthys molitrix
Source: PLoS One. 2022 Sep 16;17(9):e0274734. doi: 10.1371/journal.pone.0274734 (PMC9480980; doi:10.1371/journal.pone.0274734)
Supplement: Attachment — Submitted filename: Response to the reviewers.docx [file pone.0274734.s002.docx]

**Cover Letter (Revised Manuscript)**

8/31/2022

Dear Editor of Plos One,

I am submitting a revised manuscript for consideration of publication in Plos One. Here is detail of all observations and suggestions that were addressed carefully. Moreover, the updated information could be found with track changes file.

1. Please ensure that your manuscript meets PLOS ONE's style requirements, including those for file naming.

Revised and the manuscript is updated by following the given guidelines to meet PLOS ONE's style requirements.

2. Please include your full ethics statement in the ‘Methods’ section of your manuscript file. In your statement, please include the full name of the IRB or ethics committee who approved or waived your study, as well as whether or not you obtained informed written or verbal consent. If consent was waived for your study, please include this information in your statement as well.

Revised and updated please see the track changes in materials and methods.

3. Please ensure that you refer to Figure 1 in your text as, if accepted, production will need this reference to link the reader to the figure.

Revised and updated please see the track changes in growth performance results.

4. Please include captions for your Supporting Information files at the end of your manuscript, and update any in-text citations to match accordingly. Please see our Supporting Information guidelines for more information: <http://journals.plos.org/plosone/s/supporting-information>.

Additional Editor Comments:

The work is interesting. Relevant results have been obtained with a perspective for implementation in practice. I have some observations to the authors.

1. The toxic effects of selenium should be discussed. As well as the effects of insufficient selenium intake by the population should be discussed. <https://doi.org/10.3390/antiox11081572>

The relevant information regarding the effects of Se overdosage, and deficiency is included in the introduction section please see the tract changes in the second paragraph.

2. Author should provide a justification of choosing selenium levels of 0 mg/kg (0.0),0.3 mg/kg (0.3), 0.6 mg/kg (0.6), 0.9 mg/kg, (0.9) and 1.2 mg/kg.

This work is from a Ph.D. thesis that contained a series of experiments. In the first experiment, published in aquaculture reports, as detailed in the introduction we evaluated three Se sources () and two levels (0.5 and 1 mg/kg of the diet). As results predicted Se methionine as the best source, we aimed this experiment to narrow down the requirements by selecting 0 mg/kg (0.0),0.3 mg/kg (0.3), 0.6 mg/kg (0.6), 0.9 mg/kg, (0.9) and 1.2 mg/kg levels. Moreover, literature also in support of these dietary levels as similar levels of nano-selenium was investigated in grass carp Ctenopharyngodon idella fed with a high-fat diet. Here is a link for detail https://doi.org/10.1111/anu.13016

3. The conclusion should be written in more detail and reflect the results obtained in the study.

Revised and updated please see the track changes in the conclusion section.

Reviewers' comments:

Reviewer's Responses to Questions

Comments to the Author

1. Is the manuscript technically sound, and do the data support the conclusions?

The manuscript must describe a technically sound piece of scientific research with data that supports the conclusions. Experiments must have been conducted rigorously, with appropriate controls, replication, and sample sizes. The conclusions must be drawn appropriately based on the data presented.

Reviewer #1: Yes

2. Has the statistical analysis been performed appropriately and rigorously?

Reviewer #1: Yes

3. Have the authors made all data underlying the findings in their manuscript fully available?

The PLOS Data policy requires authors to make all data underlying the findings described in their manuscript fully available without restriction, with rare exception (please refer to the Data Availability Statement in the manuscript PDF file). The data should be provided as part of the manuscript or its supporting information, or deposited to a public repository. For example, in addition to summary statistics, the data points behind means, medians and variance measures should be available. If there are restrictions on publicly sharing data—e.g. participant privacy or use of data from a third party—those must be specified.

Reviewer #1: Yes

4. Is the manuscript presented in an intelligible fashion and written in standard English?

PLOS ONE does not copyedit accepted manuscripts, so the language in submitted articles must be clear, correct, and unambiguous. Any typographical or grammatical errors should be corrected at revision, so please note any specific errors here.

Reviewer #1: Yes

5. Review Comments to the Author

Please use the space provided to explain your answers to the questions above. You may also include additional comments for the author, including concerns about dual publication, research ethics, or publication ethics. (Please upload your review as an attachment if it exceeds 20,000 characters)

Reviewer #1:

Comment #1: Abstract should be more informative

Revised according to required information please find the track changes in the abstract section
Comment #2: Revise the English writing

Revised and Grammarly software was used to detect grammar mistakes.
Comment #3: The introduction section suggested to summarized

Revised, updated, and summarized. Please see the track changes.
Comment #4: In case of data in tables, decimal should be the same

Revised, and updated, Please check all the tables.
Comment #5: The keywords should have different words from the title

Thank you for enhancing our knowledge about it. We revised and updated please according to your worthy suggestions please find.
Comment #6: The authors should update the list of references to contain recent publications in 2020 and 2021
Comment #7: The manuscript needs thorough English proofreading for grammatical mistakes and run-on sentences.

Revised and Grammarly software was used to detect grammar mistakes.
Comment #8: When write fish name for the first time, add scientific name along with common name.

Please check it at the last of the abstract section line 14-15.
Comment #8: title suggested to be revised

Revised please find the track changes

Comment #9: Line 28: revise the written

Revised please find the track changes
Comment #10 provide weight as mean = - S.D

Initial body weight and lengths are updated in both abstract as well as material method mean with S.D. Please check lines 16, 81-82.
Comment #11: in abstract results must be written with statistical view and P value should be provided

Revised according to required information please find the track changes in the abstract section
Comment #11: better conclusion must be proved at the end of abstract

Revised according to required information please find the track changes in the abstract section
Comment #12-line 90 , provide reference
Comment #15: Line 33: average fish weight ± SD or SE

Revised and corrected ± SD please find the track changes
Comment #16: Line 55-61: This part is unnecessary, the authors should consider to revise it

Revised and updated please check the track changes.
Comment # 17: The up to date references would make the discussion part better.

Updated and some more recent references are included in the introduction and discussion sections. Please see the track changes.

Comment #18: The authors should follow the journal format.

Revised and updated please check it.

Thank you very much for your consideration.

Yours Sincerely,

Maida Mushtaq

Department of Fisheries and Wildlife, Faculty of Fisheries and Wildlife, University of Veterinary and Animal Sciences, Lahore 54000, Pakistan

Email: maida.ch17@gmail.com
